# Expanding a tyrosyl-tRNA synthetase assay to other aminoacyl-tRNA synthetases

**DOI:** 10.1016/j.dib.2015.05.021

**Published:** 2015-06-10

**Authors:** Charles J. Richardson, Eric A. First

**Affiliations:** Department of Biochemistry and Molecular Biology, Louisiana State University Health Sciences Center in Shreveport, 1501 Kings Highway, Shreveport, LA 71130, USA

**Keywords:** Aminoacyl-tRNA synthetase, High-throughput assay, Editing domain, Cyclodipeptide synthase, d-tyrosyl-tRNA deacylase

## Abstract

Aminoacyl-tRNA synthetases catalyze the attachment of amino acids to their cognate tRNAs. In general, aminoacyl-tRNA synthetase assays require stoichiometric amounts of tRNA, which limits their sensitivity while increasing their cost. This requirement for stoichiometric amounts of tRNA can be alleviated if the aminoacyl-tRNA product is cleaved following the tRNA aminoacylation reaction, regenerating the free tRNA substrate. This data article is related to the research article entitled “A continuous tyrosyl-tRNA synthetase assay that regenerates the tRNA substrate” in which this approach is used to develop a continuous spectrophotometric assay for tyrosyl-tRNA synthetase [1]. Here we present enzymes that can be used to cleave the aminoacyl-tRNA product for at least 16 of the 20 naturally occurring amino acids. These enzymes can be used to extend the tyrosyl-tRNA synthetase assay to other aminoacyl-tRNA synthetases.

## 1 Specifications table

**Table:** 

Subject area	Biology
More specific subject area	Enzyme kinetics, high-throughput drug screen
Type of data	Table
How data was acquired	Literature search
Data format	Raw text files
Experimental factors	Not applicable
Experimental features	Identities of aminoacyl-tRNA synthetases containing cis editing domains, proteins capable of trans-editing of misacylated tRNAs, cyclodipeptide synthases, and variants of these proteins that have extended substrate specificities
Data source location	Shreveport, LA, USA
Data accessibility	Data are available here with this article

### 1.1 Value of the data

• Provides a blueprint for designing high-throughput assays for aminoacyl-tRNA synthetases
• Identifies editing domains and other proteins that can be used to regenerate free tRNA from the aminoacyl-tRNA product, decreasing the cost and increasing the sensitivity of aminoacyl-tRNA synthetase assays
• Facilitates the development of high throughput screens for inhibitors of at least 16 of the 20 naturally occurring aminoacyl-tRNA synthetases

## 2 Data, experimental design, materials and methods

**Fig. 1:**
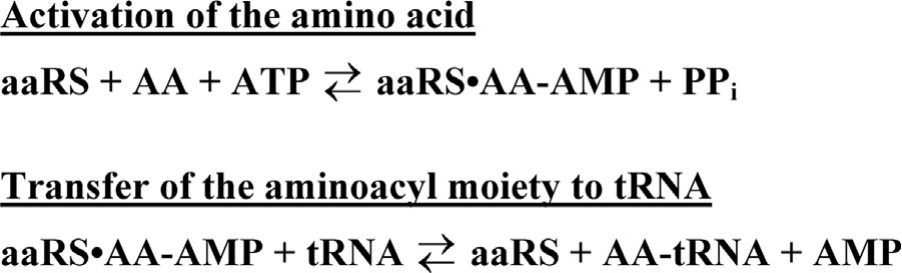
Two step reaction mechanism for the aminoacylation of tRNA. The amino acid activation and subsequent transfer of the activated aminoacyl moiety to tRNA is shown. ‘aaRS’, AA, and PP_i_ represent aminoacyl-tRNA synthetase, amino acid, and inorganic pyrophosphate, respectively. Noncovalent interactions are indicated by ‘•’ and covalent bonds are indicated by ‘-’.

Aminoacyl-tRNA synthetases (aaRSes) are essential enzymes that catalyze the attachment of amino acids to their cognate tRNAs using a two-step mechanism (Fig. 1). In the first step, the amino acid is activated by ATP, forming an enzyme-bound aminoacyl-adenylate intermediate (aaRS•AA-AMP). In the second step of the reaction, the activated aminoacyl-moiety is transferred to the 3׳ end of the cognate tRNA, resulting in the release of the aminoacyl-tRNA and AMP products.

We have developed a continuous spectrophotometric assay for one of the aminoacyl-tRNA synthetases, tyrosyl-tRNA synthetase, in which the release of AMP is coupled to the production of NADH via AMP deaminase (which converts AMP to IMP) and IMP dehydrogenase (which couples the reduction of NAD^+^ to the conversion of IMP to XMP). As the production of NADH is associated with an increase in absorbance at 340 nm, the aminoacylation of tRNA^Tyr^ by tyrosine can be monitored spectrophotometrically. In contrast to other aminoacyl-tRNA synthetase assays, where tRNA is the limiting substrate, in the tyrosyl-tRNA synthetase assay, the Tyr-tRNA^Tyr^ product is cleaved, regenerating the tRNA^Tyr^ substrate. This results in a substantial increase in the sensitivity of the assay, while significantly decreasing its cost. We have demonstrated that the tyrosyl-tRNA synthetase assay can be used to monitor the aminoacylation of tRNA by either l- or d-tyrosine, with cyclodityrosine synthase and d-tyrosyl-tRNA deacylase being used to cleave the l-Tyr-tRNA and d-Tyr-tRNA products, respectively. A detailed description of this assay can be found in [1].

In order to extend the tyrosyl-tRNA synthetase assay to other aminoacyl-tRNA synthetases, we have identified aminoacyl-tRNA synthetase editing domains, trans-editing proteins, and cyclodipeptide synthases that can be used to cleave specific aminoacyl-tRNA products. In addition, based on published literature, we have identified variants of editing domains and proteins that increase the number of different aminoacyl-tRNAs that the editing domains and proteins can cleave. This allows them to regenerate the tRNA substrate for several different aminoacyl-tRNA synthetases.

**Fig. 2:**
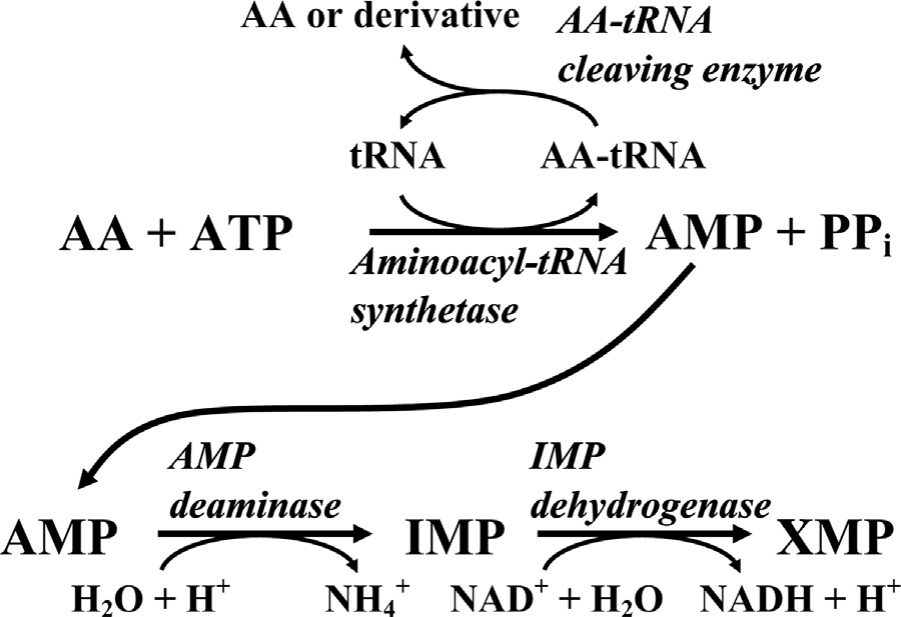
Reaction scheme for the aminoacyl-tRNA synthetase assay. The aminoacylation of tRNA is monitored by coupling the release of AMP to the production of NADH via the actions of AMP deaminase and IMP dehydrogenase. Under conditions where tRNA aminoacylation is the rate-limiting step (i.e., at sufficiently low concentrations of the aminoacyl-tRNA synthetase), the rate of the reaction will correspond to the increase in absorbance at 340 nm with respect to time. Cleavage of the aminoacyl-tRNA (AA-tRNA) product by an aminoacyl-tRNA editing protein or cyclodipeptide synthase, regenerates the unliganded tRNA, preventing it from being the limiting substrate in the reaction. AMP, IMP, XMP, PP_i_, and AA represent adenosine 5′-monophosphate, inosine 5′-monophosphate, xanthine 5′-monophosphate, inorganic pyrophosphate, and amino acid, respectively.

The basic aminoacyl-tRNA synthetase assay is shown in Fig. 2. Aminoacylation of tRNA results in the release of the aminoacyl-tRNA product, AMP, and inorganic pyrophosphate (PP_i_). We have coupled the production of AMP to the reduction of NAD^+^, allowing the assay to be followed by monitoring changes in absorbance at 340 nm (ε_340_^NADH^=6220 M^−1^ cm^−1^). Alternatively, the reaction can be followed by using inorganic pyrophosphatase to cleave the inorganic pyrophosphate product and monitoring the resulting production of phosphate (e.g. via reaction with malachite green and ammonium molybdate [2,3]). Cleavage of the aminoacyl-tRNA product is achieved by using an editing domain, trans-editing protein, or cyclodipeptide synthase that is specific for each particular aminoacyl-tRNA, or by using the M129K variant of d-tyrosyl-tRNA deacylase, which is proposed to catalyze the hydrolysis of both l- and d-aminoacyl-tRNAs and has a broad specificity with respect to the aminoacyl moiety (Table 1). In the event that a particular editing domain cannot be isolated in its active form, a variant of the full length aminoacyl-tRNA synthetase in which the synthetic site has been inactivated can be used to hydrolyze the aminoacyl-tRNA product.

## Figures and Tables

**Table 1:** Proteins that regenerate free tRNA.

**Protein**^a^	**Substrate specificity**^b^	**Literature references**
IleRS editing domain^c^	l-Val-tRNA	[4,5]
ValRS editing domain^c^	l-Thr-tRNA	[4]
LeuRS editing domain^c^	l-Ile-tRNA, l-Met-tRNA, l-Nva-tRNA	[6,7]
LeuRS-T252A editing domain^c^	l-Ile-tRNA, l-Met-tRNA, l-Nva-tRNA,	[8,9]
	l-Leu-tRNA	
ThrRS editing domain^c^	l-Ser-tRNA	[10]
ProRS editing domain^c^	l-Ala-tRNA	[11]
PheRS editing domain^c^	l-Tyr-tRNA	[12]
Ybak protein^d^	l-Cys-tRNA	[13]
ProXP-Ala protein^d^	l-Ala-tRNA	[14]
AlaX protein^d^	l-Ser-tRNA, Gly-tRNA	[15]
PrdX protein^d^	l-Ala-tRNA	[15]
ThrRS-ed protein^d^	l-Ser-tRNA	[16]
D-tyrosyl-tRNA deacylase (DTD)^d^	d-aminoacyl-tRNA	[17,18]

D-tyrosyl-tRNA deacylase M129K variant (DTD-M129K)^e^	l-Ala-tRNA, l-Asp-tRNA, l-Arg-tRNA,	[19]
	l-Cys-tRNA, Gly-tRNA, l-Glu-tRNA, l-Leu-tRNA,	
	l-Phe-tRNA, l-Pro-tRNA, l-Ser-tRNA,	
	l-Thr-tRNA, l-Tyr-tRNA, l-Val-tRNA	
AlbC^f^	l-Phe-tRNA	[20]
Cyclodityrosine synthase^f^	l-Tyr-tRNA	[21]
YvmC^f^	l-Leu-tRNA	[22]

a aminoacyl-tRNA synthetases are abbreviated using the 3-letter code for the amino acid, followed by ‘RS’.

b standard 3-letter codes are used for each aminoacyl-tRNA, Nva is the 3-letter code for norvaline.

c indicates the activity of the editing domain in the protein.

d indicates a naturally occurring trans-editing protein.

e hydrolysis of l-aminoacyl-tRNAs is assumed based on the ability of the DTD-M129K variant to bind the free l-amino acids.

f indicates a cyclodipeptide synthase, which releases the free tRNA along with a cyclic dipeptide.
